# Hydrolytic Fate of 3/15-Acetyldeoxynivalenol in Humans: Specific Deacetylation by the Small Intestine and Liver Revealed Using in Vitro and ex Vivo Approaches

**DOI:** 10.3390/toxins8080232

**Published:** 2016-07-28

**Authors:** El Hassan Ajandouz, Stéphane Berdah, Vincent Moutardier, Thierry Bege, David Jérémie Birnbaum, Josette Perrier, Eric Di Pasquale, Marc Maresca

**Affiliations:** 1 Aix Marseille Univ, CNRS, Centrale Marseille, iSm2, Marseille, France; el-hassan.ajandouz@univ-amu.fr (E.H.A.); josette.perrier@univ-amu.fr (J.P.); 2 Aix Marseille Univ, Laboratoire de Biomécanique Appliquée, UMRT24 IFSTTAR, Faculté de médecine secteur Nord, Boulevard Pierre Dramard, F-13916 Marseille Cedex 20, France; stephane.berdah@ap-hm.fr (S.B.); vincent.moutardier@univ-amu.fr (V.M.); thierry.bege@ap-hm.fr (T.B.); david.birnbaum@ap-hm.fr (D.J.B.); 3 Aix Marseille Univ, CNRS, CRN2M, Marseille, France & CSO@MyEnterix, Marseille, France; eric.di-pasquale@myenterix.com

**Keywords:** mycotoxin, deoxynivalenol, 3-acetyldeoxynivalenol, 15-acetyldeoxynivalenol, carboxylesterase, human explants, Caco-2, T84, HepG2, A498

## Abstract

In addition to deoxynivalenol (DON), acetylated derivatives, i.e., 3-acetyl and 15-acetyldexynivalenol (or 3/15ADON), are present in cereals leading to exposure to these mycotoxins. Animal and human studies suggest that 3/15ADON are converted into DON after their ingestion through hydrolysis of the acetyl moiety, the site(s) of such deacetylation being still uncharacterized. We used in vitro and ex vivo approaches to study the deacetylation of 3/15ADON by enzymes and cells/tissues present on their way from the food matrix to the blood in humans. We found that luminal deacetylation by digestive enzymes and bacteria is limited. Using human cells, tissues and S9 fractions, we were able to demonstrate that small intestine and liver possess strong deacetylation capacity compared to colon and kidneys. Interestingly, in most cases, deacetylation was more efficient for 3ADON than 15ADON. Although we initially thought that carboxylesterases (CES) could be responsible for the deacetylation of 3/15ADON, the use of pure human CES1/2 and of CES inhibitor demonstrated that CES are not involved. Taken together, our original model system allowed us to identify the small intestine and the liver as the main site of deacetylation of ingested 3/15ADON in humans.

## 1. Introduction

Mycotoxins are secondary metabolites produced by various molds infecting plants and crops and belonging chiefly to *Aspergillus*, *Penicillium* or *Fusarium* species [1,2,3,4,5]. Among the large number of existing mycotoxins (i.e., more than 350 [6]) some, such as deoxynivalenol (DON), have attracted special attention due to their prevalence and toxicity [7,8]. DON belongs to a family of mycotoxins called trichothecenes. Trichothecenes are small sesquiterpenoids with an epoxide group at position 12–13 that is involved in their toxic effect through their binding to ribosomes. This binding causes a ribotoxic effect characterized by the activation of various protein kinases, the modulation of gene expression, leading eventually to alteration of cell functions and cell toxicity [8,9,10]. Thus, exposure to DON is associated to alterations of the intestinal, immune, and brain cell functions in animals and humans [8,10,11,12,13,14,15,16].

**Figure 1:**
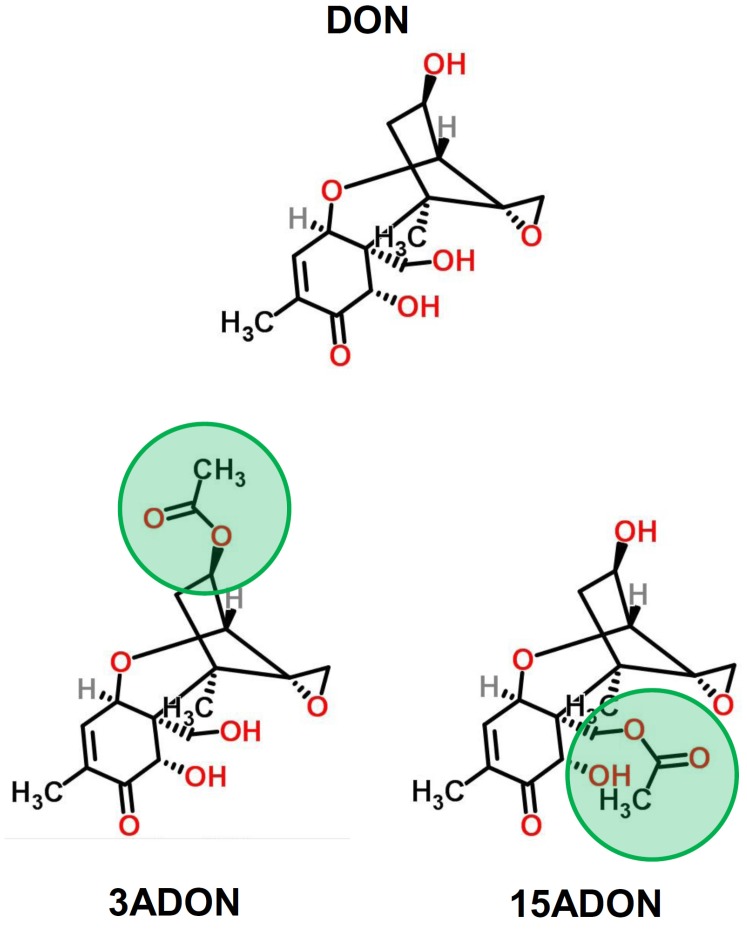
Chemical structure of DON (deoxynivalenol), 3ADON (3-acetyldeoxynivalenol) and 15ADON (15-acetyldeoxynivalenol). Green circles indicate the acetyl group on carbon 3 or 15 in 3- and 15ADON, respectively. Structures were drawn using ChemSpider 2D Image software.

In addition to native/unmodified mycotoxins, food and feeds also contain mycotoxin derivatives that may represent a risk for animals and humans, their metabolic fate and toxicity being not always characterized [4,17,18,19]. Although new molecules are regularly described, the major derivatives of DON present in food and feed correspond to fungal (i.e., the acetylated derivatives: 3- and 15-acetylDON or 3ADON and 15ADON) and plant (i.e., 3-*O*-glucoside-DON or D3G) derivatives [17,19]. 3/15ADON (Figure 1) are precursors in the synthesis of DON [20] and are thus found in food as a mixture with DON. Indeed, like DON, 3/15ADON have a high prevalence in cereals (87% and 73% of positive samples for 3ADON and 15ADON, respectively) with contamination levels ranging from 300 to 1000 μg/kg leading to daily exposure of animals and humans to 3/15ADON [21,22,23]. Although in vivo and in vitro experiments have demonstrated that 3/15ADON could be absorbed by the intestinal epithelium, oral exposures of chickens, rats and pigs to 3/15ADON only result in DON and/or detoxification products of DON (i.e., the 3/15-glucuronic acid conjugates of DON and the de-epoxide form of DON, i.e., DOM-1) being present in their blood suggesting a pre-systemic and/or systemic deacetylation [21,24,25,26,27,28].

Broekaert et al. [21] elegantly showed that both oral and intravenous (i.v) exposures of pigs to 3- or 15ADON resulted in their complete transformation into DON, demonstrating that pre-systemic and systemic deacetylation may occur in parallel. Interestingly, the authors also demonstrated that traces of 15ADON are present in the blood of chickens after oral or i.v treatment, suggesting that, at least in chickens, contrarily to 3ADON, 15ADON may partially resist deacetylation [21].

**Figure 2:**
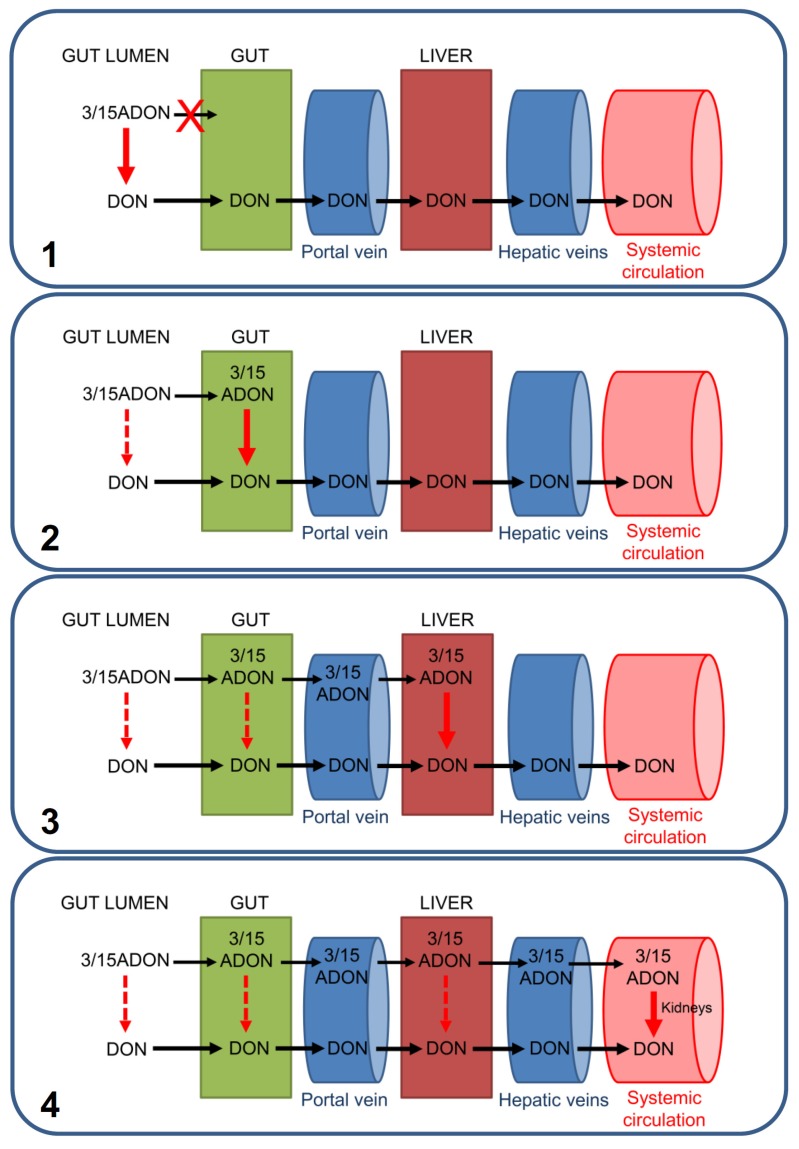
Current hypotheses regarding the deacetylation of ingested 3/15ADON. As described in the text, four hypotheses could explain animal results showing the absence of systemic 3/15ADON in case of oral exposure. (**1**) 3/15ADON may not be absorbed directly by intestinal epithelial cells (IEC), requiring their luminal transformation (by digestive enzymes and/or bacteria) prior to cross the intestinal wall. (**2**) IEC may be able to absorb 3/15ADON and to convert them in DON intracellularly, resulting in only DON being released in the portal vein linking the gut to the liver. (**3**) Part or all of intestinally absorbed 3/15ADON may reach the liver through the portal vein and may be subjected to hepatic deacetylation before release into the hepatic veins and the systemic blood circulation. (**4**) Although highly unlikely based on experimental results, part or all of the intestinally absorbed 3/15ADON may reach intact the systemic blood and be converted into DON in the kidneys (or others tissues). None of these hypotheses exclude the others, and a mix of them is possible.

The different hypotheses explaining the pre-systemic and/or systemic deacetylation of 3/15ADON are represented in Figure 2.

The first hypothesis is that 3/15ADON may not be directly absorbed by intestinal epithelial cells (IEC) and require deacetylation by gut bacteria or by luminal intestinal enzymes prior to their absorption. This hypothesis seems highly unlikely since it is known that: (i) in monogastric animals (such as humans, rats and pigs) most of the absorption of DON (and 3/15ADON) occurs fast (less than an hour) at the level of the duodenum and jejunum, where only few bacteria are present; and (ii) in vitro experiments have shown that 3/15ADON could efficiently be absorbed by the IEC [8,21,25]. Thus, it seems more plausible that without the need of a luminal deacetylation, 3/15ADON are absorbed by the IEC. Absorbed 3/15ADON may be then deacetylated intracellularly by the IEC that possess intracellular carboxylesterase (i.e., CES2) [8,29,30,31] potentially able to transform 3/15ADON in DON before its release into the blood. Alternatively, as suggested by the in vivo study conducted in pigs and chickens showing that i.v injection resulted in a fast and complete transformation of 3- and 15ADON into DON, deacetylation may take place in the liver and/or the kidneys [21]. In that case, part or all of the intestinally absorbed 3/15ADON may cross intact the intestinal wall and reach the liver where they may be deacetylated by the hepatic carboxylesterase (i.e., CES1) [8,29,30,31] and/or the kidneys that, like the intestine, express a high level of CES2 [30,31].

Although not proved, the absence of 3- and 15ADON in human blood and urine suggests that humans, like pigs, are able to completely transform 3/15ADON into DON either through pre-systemic and/or systemic mechanisms [8].

To test this hypothesis, in the present work, we used in vitro and ex vivo approaches to evaluate the deacetylation activity of the different enzymes, bacteria and cells/tissues present in humans on the way from the food matrix to the systemic blood in order to estimate the role played by each of them in the transformation of 3/15ADON into DON. Our original model systems allowed us to identify the small intestinal and hepatic epithelium as the main sites of 3/15ADON deacetylation in humans.

## 2. Results

### 2.1. Luminal Digestive Enzymes and Fecal Bacteria Possess Limited Activity on 3/15ADON

**Figure 3:**
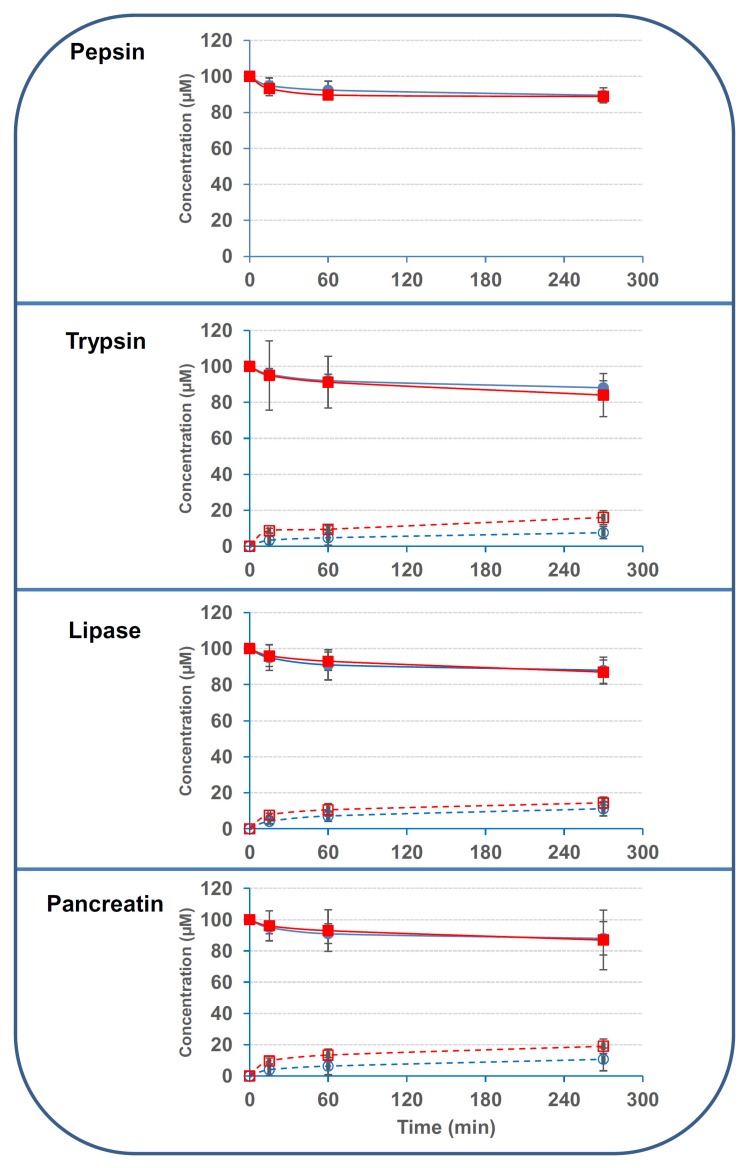
Luminal digestive enzymes possess minor activity on 3/15ADON. 3- or 15ADON (initially 100 μM) were incubated with pepsin, lipase, trypsin or pancreatin as explained in the Materials and Methods. Deacetylation of 3/15ADON and formation of DON were monitored using High Performance Liquid Chromatography (HPLC). Closed and open blue circles correspond to 3ADON disappearance and DON formation from 3ADON, respectively. Closed and open red squares correspond to 15ADON disappearance and DON formation from 15ADON, respectively (*n* = 3). Results are expressed as means ± S.D.

**Figure 4:**
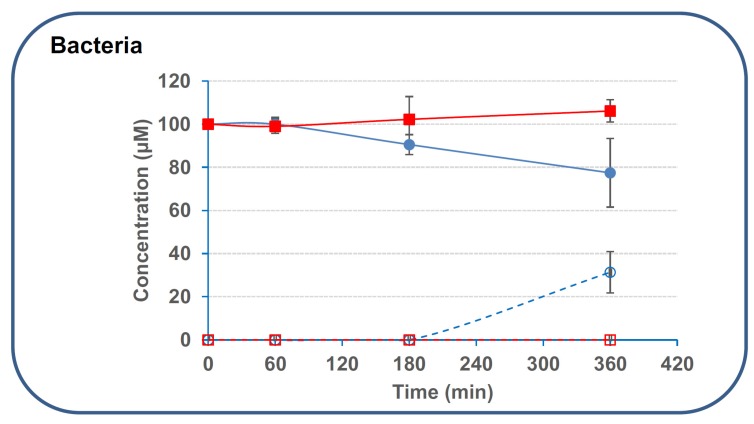
Deacetylation of 3/15ADON by human fecal bacteria. 3- or 15ADON (initially 100 μM) were incubated with human fecal bacteria as explained in the Materials and Methods. Deacetylation of 3/15ADON and formation of DON were monitored using HPLC. The symbols are the same as in Figure 3 (*n* = 3). Results are expressed as means ± S.D.

We first evaluated the capacity of secreted digestive enzymes present in the upper part of the intestinal tract to deacetylate 3/15ADON. Results showed that pepsin and pancreatic enzymes (pancreatin, trypsin, lipase) possessed very limited activity on 3- and 15ADON (around 10%–15% of deacetylation after 210–270 min incubation at 37 °C) (Figure 3). Increasing further the incubation time did not significantly improve the deacetylation, only 15%–20% of the initial concentration of 3/15ADON (100 μM) being hydrolyzed after 24 h of incubation at 37 °C (data not shown). Similarly, fecal bacteria obtained from human donors displayed no activity on 15ADON and limited activity on 3ADON with only 22.6% ± 15.9% of deacetylation after 6 h incubation (Figure 4).

As initially demonstrated by Sundstøl Eriksen and Pettersson [32], 48 h incubation with human fecal bacteria resulted in a higher deacetylation, with 82.1% ± 21.9% and 14.9% ± 3.2% of deacetylation for 3ADON and 15ADON, respectively. Overall, the specific activity of 3ADON deacetylation by secreted digestive enzymes and fecal bacteria was comprised between 24 and 57 pmol·min^−1^·mg^−1^ based on 3ADON disappearance and between 20 and 37 pmol·min^−1^·mg^−1^ based on the appearance of DON. The intervals for 15ADON were 6–94 pmol·min^−1^·mg^−1^ and 14–99 pmol·min^−1^·mg^−1^ based on 15ADON disappearance and DON formation, respectively.

### 2.2. Small Intestinal and Hepatic Human Cells Display Stronger Deacetylation Activity on 3/15ADON Than Colonic or Renal Cells

**Figure 5:**
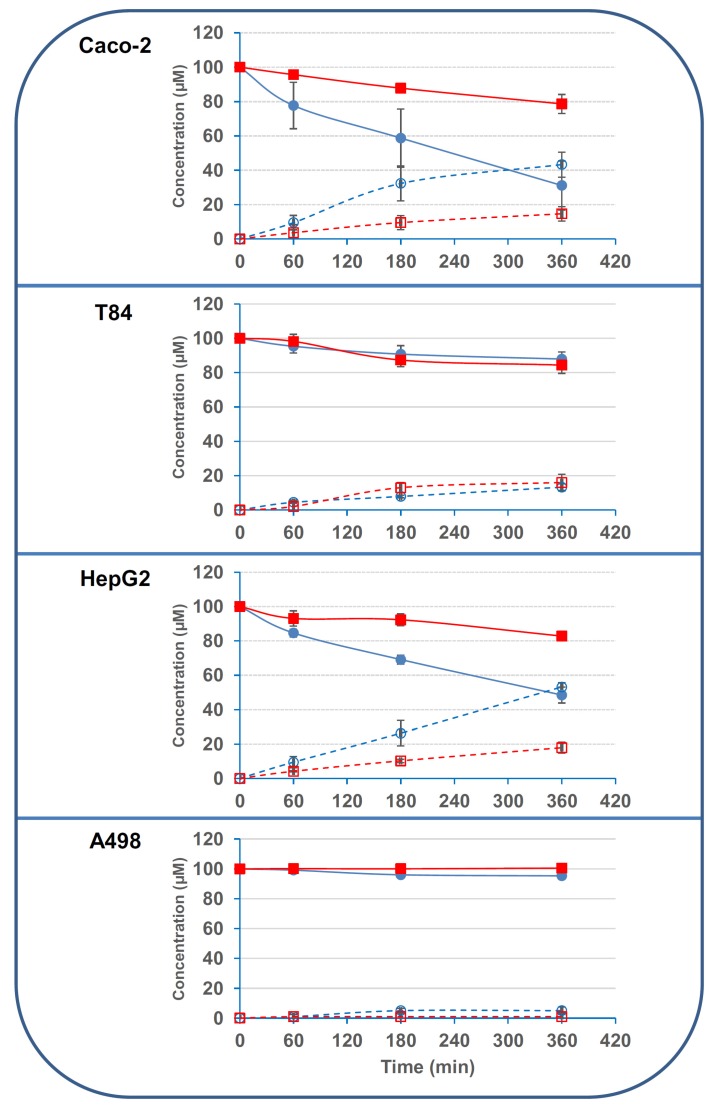
Deacetylation of 3/15ADON by human cell lines. 3- or 15ADON (initially 100 μM) were incubated with human cell lines as explained in the Materials and Methods. Deacetylation of 3/15ADON and formation of DON were monitored using HPLC. The symbols are the same as in Figure 3 (*n* = 3). Results are expressed as means ± S.D.

We then tested the deacetylation activity of human cell lines classically used to mimic the small intestine (Caco-2 cells), the colon (T84 cells), the liver (HepG2 cells) or the kidney (A498 cells). Results showed that Caco-2 and HepG2 cells were able to efficiently deacetylate 3ADON with 69.1% ± 14.3% and 52.1% ± 4.5% of 3ADON being deacetylated after 6 h incubation with Caco-2 and HepG2 cells, respectively (Figure 5). Interestingly, the activity of these cells on 15ADON was much lower, only 22.4% ± 5.5% and 16.8% ± 2.4% of 15ADON being deacetylated after 6 h incubation with Caco-2 and HepG2 cells, respectively. T84 and A498 cells displayed very limited activity on 3- and 15ADON, with only 13%–16% and 0%–5% deacetylation after 6 h incubation with T84 and A498 cells, respectively.

Based on the disappearance of 3ADON, specific activities were found to be equal to 479, 902, 80 and 79 pmol·min^−1^·mg^−1^ for Caco-2, HepG2, T84 and A498 cells, respectively. In the case of 15ADON, based on disappearance rates, specific activities were found to be equal to 152, 295, 98 and 0.4 pmol·min^−1^·mg^−1^ for Caco-2, HepG2, T84 and A498 cells, respectively. Similar results were obtained if the specific activities were determined based on the appearance of DON. Thus, human small intestinal and hepatic cells were 6 to 11-times more active on 3ADON than colonic and renal cells. The difference between the cell lines was lower in the case of 15ADON, with small intestinal and hepatic cells being 1.5 to 3-times more active than colonic cells, while renal cells were virtually inactive on 15ADON.

**Figure 6:**
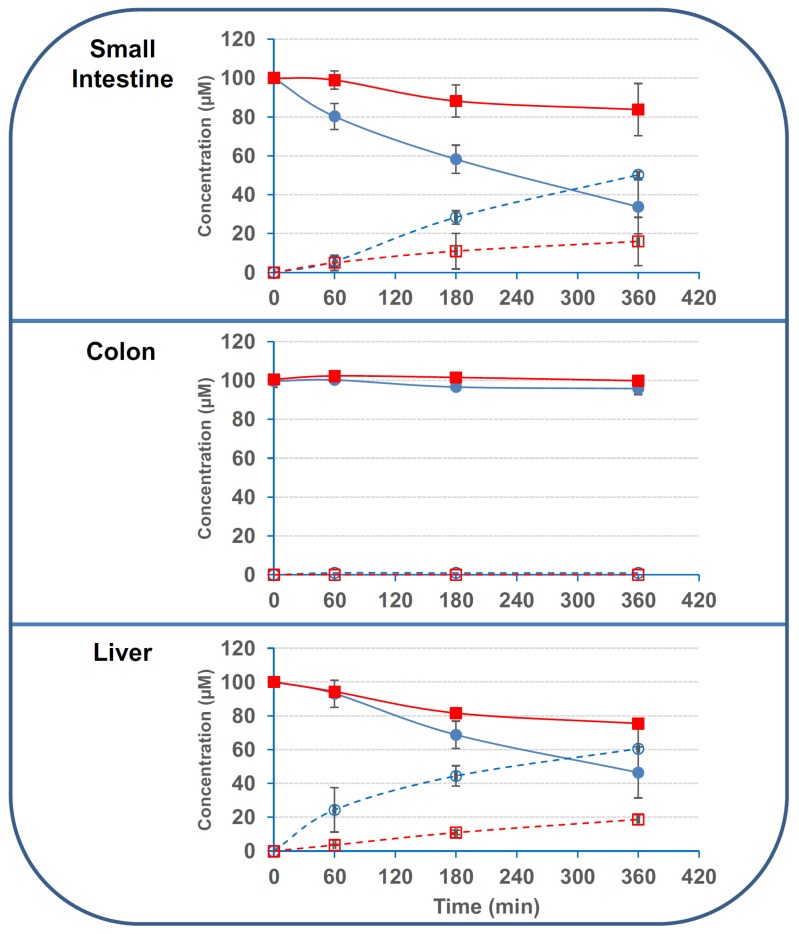
Deacetylation of 3/15ADON by human explants. 3- or 15ADON (initially 100 μM) were incubated with human explants as explained in the Materials and Methods. Deacetylation of 3/15ADON and formation of DON were monitored using HPLC. The symbols are the same as in Figure 3 (*n* = 3). Results are expressed as means ± S.D.

In order to confirm the observations made with human cell lines, we tested the activity of human tissue explants obtained from human intestinal and hepatic resections. Regarding 3ADON, we found stronger activity of small intestinal and hepatic tissues compared to colonic one, with 67.3% ± 13.8%, 54.6% ± 15.4% and 4.2% ± 3.1% of deacetylation after 6 h incubation with small intestinal, hepatic and colonic explants, respectively (Figure 6). As observed with the human cell lines, deacetylation activity of the human tissues were lower on 15ADON, with 17.2% ± 13.4%, 24.6% ± 2.8% and 0.2% ± 1.2% of deacetylation after 6 h incubation with small intestinal, hepatic and colonic explants, respectively. Based on the disappearance of 3ADON, specific activities of 714, 338 and 22.5 pmol·min^−1^·mg^−1^ were found for hepatic, small intestinal and colonic explants, respectively. For 15ADON deacetylation, specific activities were 330, 85 and 4.5 pmol·min^−1^·mg^−1^ for hepatic, small intestinal and colonic explants, respectively.

### 2.3. The Deacetylation Activity Is Present in the S9 Fractions But Is Not Due to CES1 or CES2

**Figure 7:**
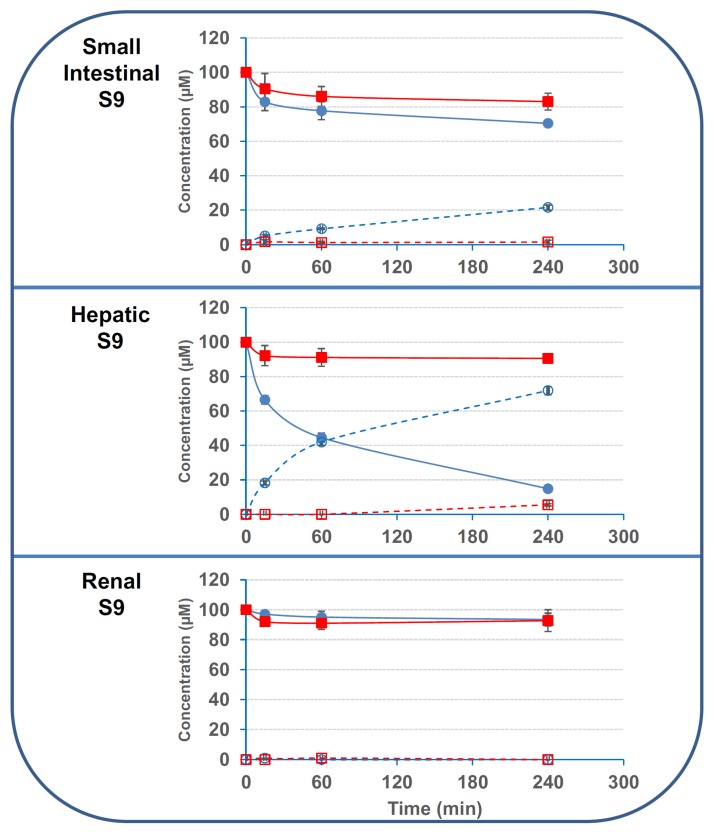
Deacetylation of 3/15ADON by human S9 fractions. 3- or 15ADON (initially 100 μM) were incubated with human S9 fractions as explained in the Materials and Methods. The symbols are the same as in Figure 3 (*n* = 3). Results are expressed as means ± S.D.

Next, we evaluated the activity of commercial pooled S9 fractions obtained from human small intestine, liver and kidneys on 3- and 15ADON. Results found with S9 fractions corroborated data obtained with human cells and tissues (Figure 7). Only small intestinal and liver S9 fractions had significant activity on 3/15ADON, whereas renal S9 fraction had minor activity, if any.

Once again, deacetylation activity of S9 fractions on 3ADON was largely higher than the activity on 15ADON with specific activities of 1250; 1791 and 33 pmol·min^−1^·mg^−1^ and 166; 208 and 16 pmol·min^−1^·mg^−1^ for intestinal, hepatic and renal S9 fractions on 3ADON and 15ADON, respectively.

Then, we evaluated the involvement of CES in 3/15ADON deacetylation. We first tested the involvement of CES in 3/15ADON deacetylation using benzil, a pan inhibitor of CES [31]. We measured the deacetylation activity of S9 fractions and human cells in the presence of benzil (100 μM). Results showed that benzil had only limited inhibitory effect on the conversion of 3/15ADON into DON by S9 fractions, i.e., 8% to 24% inhibition (Table 1).

Similarly, benzil (at 100 μM) caused a limited and none significant inhibition (i.e., 10%–20%) of the deacetylation of 3/15ADON by human cell lines (data not shown). To confirm that CES were actually inhibited by benzil, we monitored the activity of cells and S9 fractions on para-nitrophenyl acetate (pNPA), a chromogenic substrate of CES. Results showed that cell lines and S9 fractions were all able to efficiently cleave pNPA, confirming that high levels of CES were present even in the cells and S9 fractions with limited activity on 3/15ADON (i.e., A498 and T84 cells and renal S9 fraction) (Table 2).

Importantly, benzil at 100 μM caused a dramatic inhibition of the deacetylation of pNPA ranging from 83% to 96% inhibition depending of the system tested confirming its ability to inhibit CES activity (Table 2). Finally, we tested the activity of commercial recombinant human CES1 and CES2 on 3/15ADON. Although recombinant CES1 and CES2 displayed strong benzil-sensitive activity on pNPA (Table 2), we failed at observing any deacetylation activity of these enzymes on 3/15ADON, even after 24 h of incubation of 3/15ADON with 5 μg/mL of CES1/2 (data not shown).

## 3. Discussion

Numerous studies conducted in animals have shown that, although 3/15ADON are able to cross intact the gut wall, their ingestion does not lead to their presence into the systemic blood [21,26,27,28]. Similarly, although humans are daily exposed to 3/15ADON through contaminated food, analysis of human blood and urine failed at detecting 3/15ADON [8]. These two observations suggest that, once ingested, 3/15ADON are subjected to pre-systemic and/or systemic deacetylation, the site(s) of such deacetylation remaining unknown until now. In the present study, using original models, we were able to identify precisely the organs responsible for the deacetylation of 3/15ADON in humans. Table 3 summarizes the specific deacetylation activities of enzymes, cells and tissues used in this study based either on the kinetics of 3/15ADN disappearance or DON appearance.

**Figure 8:**
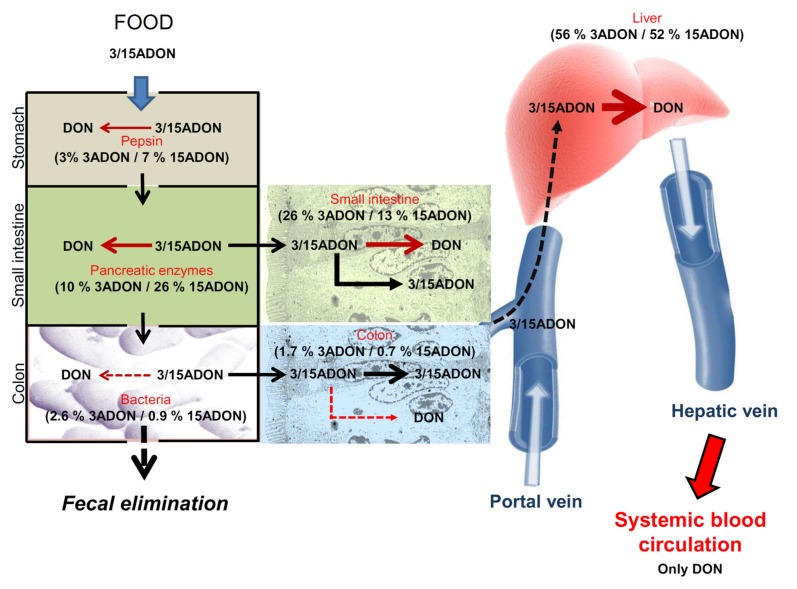
Overview of deacetylation of 3/15ADON in humans. Based on specific activities calculated in Table 3, we were able to assess the respective implication of enzymes, cells and tissues, expressed as percentage of total activity, in the deacetylation of 3- and 15ADON in humans, the contribution of each compartment being calculated considering that total 3/15-ADON deacetylation activity is the sum of specific activities of digestive enzymes, fecal bacteria, small intestinal, hepatic and colonic tissues.

Based on data of Table 3, Figure 8 proposes an overview of the hydrolytic fate of 3/15-acetyl-deoxynivalenol in humans, with the contribution of each compartment considering that total 3/15-ADON deacetylation activity is the sum of specific activities of digestive enzymes, fecal bacteria, small intestinal, hepatic and colonic tissues.

Our results showed that pepsin has 3% and 7% of total deacetylation capacity for 3- and 15ADON, respectively and that pancreatic enzymes display higher deacetylation activity with 10% and 26% of deacetylation of 3- and 15ADON, respectively. In accordance with previous publication showing 78% ± 30% deacetylation of 3ADON after 48 h incubation [32], we confirmed that human fecal bacteria are able to deacetylate 3/15ADON. However, based on specific activity, this bacterial deacetylation is very limited and only accounts for 2.6% and 0.9% of total deacetylation of 3- and 15ADON, respectively. Taken together, our results show that the intestinal luminal events play a minor role in the deacetylation of 3/15ADON accounting for 16% and 34% of deacetylation of 3- and 15ADON, more than two thirds of 3/15ADON being able to enter/cross the intestinal epithelial cells.

We find that once 3/15ADON are absorbed, the small intestine and the liver are the two major sites of 3/15ADON deacetylation, with 26% and 56% of 3ADON and 13% and 52% of 15ADON being hydrolyzed by small intestinal and hepatic tissues, respectively, colonic and renal cells playing limited roles in 3/15ADON deacetylation. Our demonstration of a strong deacetylation capacity of the liver compared to kidneys indicates that the hepatic conversion is responsible for the observed conversion of 3/15ADON into DON after i.v exposure of pigs and chickens [21]. Interestingly, although Caco-2 and T84 cells both originate from colonic adenocarcinoma, Caco-2 cells possess higher activity on 3/15ADON than T84 cells confirming, if needed, that Caco-2 cells are a valuable model of small intestinal epithelium.

We observed that deacetylation of 3ADON is higher than the one of 15ADON in Caco-2, HepG2, intestinal, colonic and liver explants, and the corresponding S9 fractions, as well as in colonic bacteria and A498 cells. However, no such discrimination between 3ADON and 15ADON is observed with the digestive enzymes, T84 cells and renal S9 fraction. Interestingly, preferential deacetylation of 3ADON compared to 15ADON has also been reported in HepG2 by others [33], in plant cells [34,35] and in vivo with chickens since exposure to 15ADON, but not 3ADON, results in some acetylated form being detected into their blood [21]. At this stage, the reason of this selectivity remains unknown but could rely on an easier access and/or higher affinity of 3ADON compared to 15ADON for the catalytic site of the enzyme(s) responsible of their deacetylation.

We tried to identify the enzyme(s) present in human intestinal and hepatic cells that are responsible for the deacetylation of 3/15ADON. Initially, based on the nature of the chemical modification, we thought that carboxylesterases may be responsible [8]. Surprisingly, our results clearly demonstrate that CES are not responsible for the deacetylation of 3/15ADON by human cells. First, although they possess strong CES activity as mentioned in the literature [29,30,31] and demonstrated with the chromogenic CES substrate para-nitrophenyl acetate (pNPA), human colonic and renal cell lines (T84 and A498 cells), human colonic explants and renal S9 fractions have no or low deacetylation activity on 3/15ADON. In addition, benzil (at 100 μM), a pan inhibitor of CES [31], strongly inhibits CES activity on pNPA (85%–96% inhibition) but moderately inhibits 3/15ADON deacetylation (8%–24% inhibition). Finally, recombinant human CES1 and CES2 were unable to cause the deacetylation of 3/15ADON confirming that these CES are not involved. Although not tested, a role of other CES such as CES3 is highly unlikely since benzil is known to inhibit all type of CES and that CES3 is present not only in hepatic but also in colonic cells that weakly hydrolyze 3/15ADON according to our results [36]. Work is underway to identify the enzyme(s) present in small intestinal and hepatic epithelial cells responsible for 3/15ADON deacetylation. Once these enzyme(s) are identified, we will potentially be able to understand the biochemical reason(s) of the preferential deacetylation of 3ADON compared to 15ADON.

In conclusion, we were able to precisely quantify the role played by the different enzymes, cells and organs in the deacetylation of 3- and 15ADON by humans and to identify the small intestinal and hepatic epithelium as major site of deacetylation of acetylated derivatives of DON. In addition to answer a specific question regarding the deacetylation of 3/15ADON in humans, this study demonstrates the high value of our original model system to evaluate and predict the fate of any contaminants present in human food.

## 4. Materials and Methods

### 4.1. Materials

Human cell lines (A498, Caco-2, HepG2, and T84) were from ATCC (Molsheim, France). Culture medium, sterile PBS (Phosphate buffer saline) and culture supplements (antibiotics, serum, trypsin-EDTA solution) were from Thermo Fisher Scientific (Illkirch, France). Cell culture plastics were from Dominique Dutscher (Saint-Maximin-la-Sainte-Baume, France). DON, 3ADON and 15ADON were from Romer labs (Tulln, Austria). Pooled S9 fractions from human small intestine, liver and kidneys were from Xenotech (Kansas City, KS, USA). Recombinant human CES1 and CES2 were from R&D Systems (Minneapolis, MN, USA). All others reagents were from Sigma-Aldrich (Saint-Quentin Fallavier, France).

### 4.2. Cell Culture

A498, Caco-2, T84 and HepG2 cells were used as models of human renal, small intestinal, colonic and hepatic cells, respectively. Cells were routinely grown in culture media (Dulbecco’s Modified Eagle Medium (DMEM), DMEM:F12 mixture and Minimum Essential Media (MEM) for A498/Caco-2, T84 and HepG2 cells, respectively) supplemented with 10% fetal calf serum and 1% antibiotics (streptomycin–penicillin solution). Cells were grown in 25 cm^2^ ventilated flask maintained at 37 °C in a 5% CO_2_ incubator. Cells were routinely passed using trypsin-EDTA solution. For deacetylation experiments, cells were seeded onto 6-well plates at an initial density of 200,000 cells/cm^2^. HepG2 and A498 were used 3–4 days post-seeding when confluence was reached. Caco-2 and T84 cells were let to differentiate for 10–14 days post-seeding before being used. In all cases, cells were washed three times with sterile phosphate buffer saline PBS^++^ (PBS containing 100 mg/L of calcium and magnesium salts) before deacetylation assay.

### 4.3. Preparation of Human Intestinal and Hepatic Explants

Human tissue samples were obtained from patients undergoing surgery at the Hospital Nord of Marseille, unit of general and gastrointestinal surgery according to a collaborative “clinical transfer” project. The procedures were approved by the French ethic committee (CODECOH n° DC-2011-1319). Patients were from both genders (six men, three women), 31–74 years of age, all of European origin. All patients agree to use their tissues for research purposes. Samples were taken from macroscopically unaffected area as identified by the surgeon for intestinal samples or the pathologist for liver resections. Diagnoses leading to surgery were carcinoma for intestine or liver. After resection, the specimens were placed in ice-cold non-sterile Krebs solution implemented and transferred to the school of medicine on the same campus within 5 min, composition of the Krebs solution being previously described [37]. The tissues were rinsed several times and maintained in oxygenated Krebs during 1 h for recovery before final dissection. Intestinal and hepatic resections were extensively washed and maintained in ice-cold culture media containing 1% streptomycin/penicillin solution and 50 μg/mL gentamycin. Tissues were cleaned from vascular vessels and conjunctive tissue using forceps under binocular microscope. In case of intestinal resection, muscularis and plexus were removed and only epithelial layer was kept. Intestinal and hepatic explants (wet weight of 20–30 mg) were then isolated from the cleaned resections using surgical punch (diameter of 0.5 cm^2^). All these operations were achieved in less than 3 h after the resections were obtained from the surgery unit. Finally, the explants were washed 3 times with PBS^++^ and transferred into 6-well plates before being used for deacetylation assay.

### 4.4. Preparation of Human Fecal Bacteria

The whole procedure involving fecal bacteria (including the use of fecal bacteria for the deacetylation assay) was performed in an anaerobic cabinet (Coy Laboratory Products, Grass Lake, MI, USA). Human fecal bacteria were isolated from three volunteers (men) working in the department, 40–50 years of age. Stools were collected fresh in a sterile container and were immediately introduced into the anaerobic cabinet. Feces samples were taken by pushing a cut-off disposal 1 mL pipette tip into the inner part of the stools. Samples were then mechanically suspended in 9 mL of degassed PBS^++^. Homogenates were allowed to decant for ten minutes to eliminate insoluble matter, and supernatants (1 mL aliquots) were transferred to sterile Eppendorf tubes and used for deacetylation assay.

### 4.5. Deacetylation Assay

Stock solutions of DON, 3ADON and 15ADON at 2 mg/mL were prepared in absolute ethanol and stored at −20 °C. For deacetylation assay, 3- or 15ADON were incubated with cells, explants, bacteria or digestive enzymes after being diluted in PBS^++^ at a final concentration of 100 μM. PBS^++^ solution containing only ethanol (at 1.5% final concentration) or DON (at 100 μM) were used in parallel as negative and positive control, respectively. For cells and explants, deacetylation assay take place in 6-well plates, wells being filled with 2 mL of PBS^++^ containing either ethanol, DON, 3- or 15ADON. For fecal bacteria and digestive enzyme assay, deacetylation assay was conducted in 2 mL Eppendorf tubes filled with 1.5 mL of PBS^++^ containing either ethanol, DON, 3- or 15ADON. In vitro intestinal digestion by luminal enzymes was performed using porcine enzymes from Sigma-Aldrich and physiological conditions [38]. Briefly, 3- or 15ADON were incubated with pepsin (at 1.6 mg/mL corresponding to 900 U/mL), trypsin (at 0.35 mg/mL corresponding to 6000 U/mL), pancreatic lipase (at 0.75 mg/mL corresponding to 18 U/mL) or pancreatin (at 1.23 mg/mL, lipase activity >10 U/mL, protease activity >100 U/mL, amylase activity >100 U/mL). Except for pepsin for which the digestion was conducted in HCl solution at 10 mM (pH 2.3), in vitro digestion was performed in PBS^++^ (pH 7.4). Finally, deacetylation assay was also performed with commercial pooled S9 fractions from human liver, small intestine and kidneys as well as with pure recombinant human CES1 and CES2. S9 fractions and CES1/2 were used at 0.1 mg/mL and 5 μg/mL (corresponding to CES activity >20 U/mL), respectively. In all cases, aliquots of the reaction solution were collected immediately (i.e., “time zero” sample) or after the indicated time period at 37 °C and analyzed by HPLC. In order to normalize the results obtained with the different cells, tissues and enzymes and to express the deacetylation activity as specific activity (pmol of 3/15ADON hydrolyzed (or pmol of DON formed) per min and per mg of protein), protein contents were measured using the Lowry’s procedure [39] and bovine serum albumin as a reference as previously described [40,41]. In all cases, the experiments were conducted in triplicate (*n* = 3).

### 4.6. HPLC Analysis

**Figure 9:**
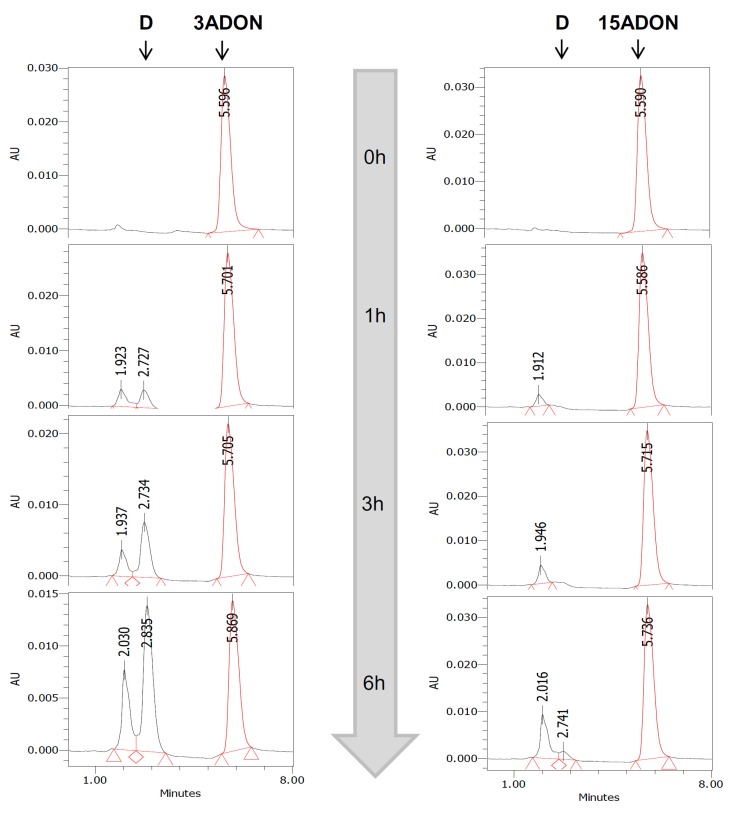
Example of HPLC analysis of the deacetylation of 3- and 15ADON. 3- or 15ADON (initially 100 μM) were incubated with HepG2 cells for 0, 1, 3 or 6 h. At the end of the incubation period, aliquots of media were analyzed using HPLC as explained in Materials and Methods. DON and 3/15ADON were clearly separated with retention times of 2.7 and 5.7 min, respectively.

Deacetylation was monitoring both in term of disappearance of 3- or 15ADON and formation of DON. Measurement of DON, 3ADON and 15ADON concentrations was performed by reversed-phase HPLC using Waters Alliance System equipped with a Waters 2690 XE separation module and a Waters 996 photodiode array detector (Waters, Saint Quentin Falavier, France). The separation was achieved by mean of a LiChroCart column (125 mm × 4 mm) packed with 5 μm RP-18 Purospher gel (Merck, Darmstadt, Germany) and equipped with a LiChroCart precolumn (4 mm × 4 mm) packed with the same material (Merck). The precolumn and column were placed in room temperature. The elution procedure was carried out at 0.7 mL·min^−1^ by a methanol–water solvent, using the following conditions: 40% methanol during 2 min followed by an increase in methanol to 50% at 2.1 min. The detection wavelength was set at 237 nm allowing the best signal/baseline ratio for simultaneous detection of the three DON forms in the incubation mediums. Under these conditions, DON and 3/15ADON were clearly separated, the retention time being around 2.7 min and 5.7 min, for DON and 3/15ADON, respectively (Figure 9). Quantification was based on calibration with the corresponding standard using Millinium Software (Waters, Saint Quentin Falavier, France).

### 4.7. Measurement of Carboxylesterases (CES) Activity

Carboxylesterase activity was measured using the chromogenic substrate para-nitrophenyl acetate (pNPA). Briefly, cells, explants, S9 fractions or recombinant CES1/2 were incubated with 1 mM of pNPA diluted in PBS^++^ at 37 °C. Color development due to the formation of para-nitrophenol (pNP) was recorded using optical density measurement at 405 nm over time. The amount of pNP formed was calculated using its extinction coefficient (18,000 M^−1^·cm^−1^). Specific activity of deacetylation of pNPA was then expressed as nmol of pNP formed per min and per mg of protein.

## Figures and Tables

**Table 1:** Effect of benzil (100 μM) on the deacetylation activity of S9 fraction on 3/15ADON. Specific activities were calculated from deacetylation kinetics and are expressed as pmol of 3/15ADON hydrolyzed (or DON formed) per minute and per mg of protein (*n* = 3). Results are expressed as means ± S.D.

Conditions	Disappearance of 3ADON	Disappearance of 15ADON	Formation of DON from 3ADON	Formation of DON from 15ADON
**S9 Intestine**	1166 ± 110	208 ± 37	916 ± 57	250 ± 54
**S9 Intestine + Benzil**	875 ± 83	171 ± 79	791 ± 6.6	191 ± 37
**S9 Liver**	1729 ± 27	166 ± 3.5	1791 ± 48	104 ± 12
**S9 Liver + Benzil**	1583 ± 39	125 ± 15	1395 ± 18	79 ± 29

**Table 2:** Effect of benzil (100 μM) on the deacetylation activity of cell lines, S9 fraction and recombinant CES1/2 on pNPA (para-nitrophenyl acetate). Specific activities were calculated from deacetylation kinetics and are expressed as nmol of pNPA hydrolyzed per minute and per mg of protein (*n* = 3). Results are expressed as means ± S.D.

Conditions	Without Benzil	With Benzil	% of Inhibition
**Caco-2**	6.2 ± 1.8	1.1 ± 0.1	83 ± 16
**T84**	6.6 ± 0.7	0.9 ± 0.2	85 ± 14
**HepG2**	15.7 ± 1.1	2.1 ± 0.1	87 ± 12
**A498**	6.2 ± 1.8	1.1 ± 0.1	83 ± 16
**S9 IntestineT84**	55 ± 9.9	8.3 ± 2.7	84 ± 15
**S9 Liver**	142 ± 4.6	21 ± 2.2	85 ± 14
**S9 Kidney**	69 ± 7.6	8.1 ± 1.4	88 ± 11
**CES1**	4965 ± 197	167 ± 10	96 ± 3.3
**CES2**	5671 ± 207	214 ± 43	96 ± 3.7

**Table 3:** Specific activity of digestive enzymes, fecal bacteria, cell lines, explants and S9 fractions on 3/15ADON. Specific activities were calculated from deacetylation kinetics and are expressed as pmol of 3/15ADON that disappeared (or pmol of DON formed) per minute and per mg of protein (*n* = 3). In the case of pepsin, the rate of formation of DON was not determined (ND) due to the presence of a contamination peak (present in the pepsin solution) at the retention time of DON. Results are expressed as means ± S.D.

Conditions	Disappearance of 3ADON	Disappearance of 15ADON	Formation of DON from 3ADON	Formation of DON from 15ADON
**Pepsin**	34 ± 7.5	40 ± 4.5	ND	ND
**Trypsin**	57 ± 19	94 ± 62	36 ± 17	99 ± 19
**Lipase**	48 ± 21	42 ± 18	34 ± 11	42 ± 8.8
**Pancreatin**	24 ± 16	26 ± 17	20 ± 15	22 ± 9.7
**Fecal bacteria**	34 ± 0.9	5.8 ± 1.3	37 ± 1.6	14 ± 0.6
**Caco-2**	479 ± 97	152 ± 38	298 ± 48	97 ± 27
**T84**	80 ± 24	98 ± 29	72 ± 11	91 ± 30
**HepG2**	902 ± 78	295 ± 41	920 ± 34	312 ± 52
**A498**	79 ± 46	0 ± 3.1	63 ± 39	0 ± 4.7
**Small intestinal explant**	338 ± 69	85 ± 65	252 ± 7.5	80 ± 62
**Colonic explant**	22 ±13	4.5 ± 5.6	14 ± 9.3	2.1 ± 4.1
**Liver explant**	714 ± 198	330 ± 34	793 ± 231	238 ± 38
**S9 Small intestine**	1250 ± 79	166 ± 20	875 ± 54	128 ± 45
**S9 Liver**	1791 ± 29	208 ± 41	1479 ± 50	125 ± 33
**S9 Kidney**	33 ± 8.3	16 ± 5.8	12 ± 16	8.5 ± 11
